# 3D Imaging of the Transparent *Mycobacterium tuberculosis*-Infected Lung Verifies the Localization of Innate Immune Cells With Granuloma

**DOI:** 10.3389/fcimb.2020.00226

**Published:** 2020-05-15

**Authors:** Gyeong-Yi Kang, Hyeong-Jun Rhyu, Hong-Hee Choi, Sung Jae Shin, Young-Min Hyun

**Affiliations:** ^1^Department of Anatomy, Yonsei University College of Medicine, Seoul, South Korea; ^2^BK21 PLUS Project for Medical Science, Yonsei University College of Medicine, Seoul, South Korea; ^3^Department of Medicine, Yonsei University College of Medicine, Seoul, South Korea; ^4^Department of Microbiology, Yonsei University College of Medicine, Seoul, South Korea; ^5^Institute for Immunology and Immunological Disease, Yonsei University College of Medicine, Seoul, South Korea

**Keywords:** granuloma, innate immunity, lung, tissue clearing, tuberculosis

## Abstract

Using a novel tissue-clearing method, we aimed to visualize the three-dimensional (3D) distribution of immune cells within *Mycobacterium tuberculosis* (Mtb)-infected mice lungs. Ethyl cinnamate-based tissue clearing of Mtb-infected mice lungs was performed to obtain transparent lung samples, which were then imaged using a light sheet fluorescence microscope. Using the 3D images, we performed quantitative analysis of the immune cell population within multiple granulomas. In addition, to compare the data from the tissue clearing method, we performed histopathological and immunofluorescence analyses, and flow cytometry. We then created 3D images of the Mtb-infected lung that successfully demonstrated the distribution of blood vessels, immune cells, and granulomas. Since the immune cells within a granuloma could be separately selected and counted, the immune cell population within a specific lesion could be quantified. In addition, macroscopic analysis, e.g., the size or shape of a granuloma, as well as microscopic analysis could be performed as intact lung samples were used. The use of the tissue clearing method in infected lungs could be a novel modality for understanding the role of the immune system in the pathogenesis of tuberculosis.

## Introduction

Tuberculosis (TB), a pulmonary disease caused by a group of infectious agents named *Mycobacterium tuberculosis* (Mtb) complex, remains one of the major health concerns worldwide. World Health Organization (WHO) reported that nearly 10.4 million people were infected, and 1.7 million deaths were caused by the contagious disease in 2016 (Floyd et al., 2018). The standard treatment for TB includes multiple anti-TB drugs, i.e., isoniazid, rifampin, ethambutol, and pyrazinamide (Tiberi et al., 2018). However, with the advent of drug-resistant TB, which exhibits resistance against one or more of the drugs in the initial regimen, chemotherapy alone has not been demonstrated to control the disease completely (Tiberi et al., 2018). Therefore, novel therapeutic approaches for TB are required.

In light of this need for a new treatment, several researches have attempted to elucidate the role of the innate immune system in the pathogenesis of the disease. The primary cells involved in the initial immune response against TB infection are macrophages, including resident alveolar and monocyte derived macrophages, and neutrophils (Schlesinger, 1996). The defense mechanism of macrophages against the infective agent is relatively well-known. As Mtb enter the alveoli, alveolar macrophages phagocytose the bacteria and subsequently produce various cytokines, recruiting monocytes from the blood and leading to mobilization of monocyte derived macrophages. This is followed by production of reactive oxygen species (ROS) and apoptosis of the recruited macrophages (Pieters, 2008). Recently, several counteractive actions of Mycobacteria against macrophages have also been found (Lerner et al., 2015). In contrast, several findings have identified a controversial role of neutrophils in regard to their interaction with the pathogen (Lowe et al., 2012). Being the most prominent immune cells in human TB, neutrophils are also the most common leukocytes in human blood (Eum et al., 2010). Animal studies have demonstrated that neutrophils play a crucial role in the initial defense against TB (Pedrosa et al., 2000; Fulton et al., 2002; Sugawara et al., 2004). Recruitment of neutrophils results in decreased colony forming units (CFU) while their depletion causes the opposite (Sugawara et al., 2004). One of the known antibacterial actions of neutrophils is its formation of a DNA scaffold, named neutrophil extracellular traps (NET) that entraps pathogens and facilitates bactericidal actions (Papayannopoulos, 2018). However, it has also been reported that there was no difference in the disease course in granulocyte-depleted mice and normal mice (Seiler et al., 2000). Additionally, the contribution of neutrophils is limited to the early stages of the disease (Petrofsky and Bermudez, 1999; Pedrosa et al., 2000; Sugawara et al., 2004; Zhang et al., 2009). In chronic TB infection, neutrophil depletion was shown to be correlated with a decrease in CFU (Zhang et al., 2009). Data from patients also suggest that higher neutrophil count is associated with worse prognosis in well-established TB (Barnes et al., 1988). To summarize, these findings indicate that the role of neutrophils in the immune response of TB remains to be clarified and studying their functions at different stages of the disease may provide a deeper understanding of the pathogenesis.

One possible approach to interpret the complicated immune reaction is to observe the spatial distribution of the immune cells. It has been reported that Mtb-infected macrophages become highly mobile and egress from the primary granuloma, consequently becoming a source of new granulomas in zebrafish and mouse (Davis and Ramakrishnan, 2009; Cohen et al., 2018). This implies that the physical distribution of Mtb-infected macrophages, or how they translocate out of the granuloma, can affect the progress of the disease. In contrast, the local influx of neutrophils has been correlated with worse prognosis while suppressing it by chemokine modulation has a better outcome (Condos et al., 1998; Nandi and Behar, 2011). These findings show that the course of the disease is closely linked with the distribution of the immune cells, such as neutrophils and macrophages. Traditionally, several modalities, e.g., hematoxylin and eosin (H&E) staining, immunohistochemistry, and fluorescence microscopy, have been used to localize cells in a pathologic condition. However, all these methods provide limited information, since the data are mostly 2D images with the possibility of omitting cells. Recently, total organ imaging enabled by a method that makes an organ transparent, namely tissue clearing, is emerging as a novel technique in various fields of research (Chung et al., 2013; Klingberg et al., 2017). One of tissue clearing methods, PACT (passive clarity technique), has also been used for fluorescence-labeled Mtb in the Mtb-infected mouse lungs (Cronan et al., 2015). In this study, we used the another tissue clearing technique using Ethyl cinnamate (ECi) to visualize immune cells within the pathologic tissue. We used 3D imaging technique of the tissue-cleared lung to localize neutrophils and macrophages in Mtb-infected mice lungs. Providing data on the spatial distribution of these immune cells and granulomas, we propose a new modality in understanding the role of the innate immune system in the pathogenesis of TB.

## Methods

### Mice

C57BL/6 mice were purchased from Japan SLC Inc. (Shizuoka, Japan). C57BL/6, CX3CR1-GFP (green fluorescent protein) (Jung et al., 2000) and LysM-GFP (Faust et al., 2000) mice were maintained under specific-pathogen-free conditions at the Avison Biomedical Research Center, Yonsei University College of Medicine. In order to minimize the unintended loss in leukocyte motility or its bactericidal function, heterogenetic (+/–) LysM-GFP and CX3CR1-GFP mice were used in the experiments. All animal experiments were performed with the approval by the animal ethics committee of Yonsei University College of Medicine (2016-0178 and 2018-0218).

### Infection of Mice

Mice were infected under strict barrier conditions in a BSL-3 facility at the Avison Biomedical Research Center, Yonsei College of Medicine. Briefly, mice were challenged with the pre-calibrated Mtb H37Rv (ATCC 27294) via aerosol using an airborne infection apparatus (Glas-Col, USA), and ~200 viable bacteria were delivered. Mice at 4 weeks post-infection were used. For the bacterial growth analysis, the lungs were homogenized, and serially diluted samples were plated onto Middlebrook 7H11 agar plates (Becton Dickinson, USA) supplemented with 10% OADC (oleic acid albumin dextrose catalase; Difco Laboratories, USA), 2 μg/mL 2-thiophenecarboxylic acid hydrazide (Sigma-Aldrich, USA), and amphotericin B (Sigma-Aldrich). The bacterial colonies were counted after 3–4 weeks of incubation period at 37°C. The animal ethics committee of Yonsei University College of Medicine approved all of the experimental protocols used (2016-0178 and 2018-0218).

### Histopathological and Immunofluorescence Studies

The naïve and Mtb-infected mice lungs were isolated and fixed in 10% formalin overnight. Fixed lungs were serially dehydrated using ethanol and then infiltrated with paraffin. The lungs were then embedded into the paraffin wax block. Paraffin blocks were sectioned by 4–5 μm thickness, and then stained using H&E. Samples were observed using Olympus BX51 microscope (Olympus, Japan). For immunofluorescence imaging, antigen retrieval was performed using Tris-EDTA. Immune cells were labeled using anti-CD3 (T cells; BioLegend, USA), anti-Ly-6G (neutrophils; Biolegend, USA), and anti-F4/80 (macrophages; BioLegend, USA) antibodies and nuclei were stained using DAPI (4',6-diamidino-2-phenylindole). The slides were observed using the LSM700 confocal microscope (Carl Zeiss, Germany). Volocity Software (Quorum Technologies, Canada) software was used for data analysis.

### Flow-Cytometry

The lung samples were minced into 2-4 mm pieces using scissors. The samples were then incubated in 3 mL of cellular dissociation buffer [RPMI medium; Biowest, France)], which contained 0.1% collagenase type IV (Worthington Biochemical Corporation, USA) and 1 mM CaCl_2_ and 1 mM MgCl_2_ for 30 min at 37°C. The dissociated cells were then filtered using a 40 μm cell strainer (BD Biosciences, USA) in RPMI medium supplemented with 2% fetal bovine serum (FBS, Biowest, France), and the erythrocytes were removed by incubating the samples in red blood cell lysis buffer (Sigma-Aldrich, USA) for 3 min at room temperature. Finally, the single cells were washed twice in RPMI medium supplemented with 2% FBS before analysis. For flow-cytometric analysis, cells were first washed with 2% FBS containing PBS and anti-CD16/32 antibodies were treated for blocking function at 4°C for 20 min. Surface molecules were labeled with fluorochrome-conjugated anti-CD45 (immune cells), anti-Siglec-F (alveolar macrophages), anti-Ly-6G (neutrophils) (BD bioscience, USA), anti-F4/80 (macrophages), anti-CD11b (monocytes/macrophages, granulocytes) (eBioscience, USA), anti-CD64 (monocyte-derived macrophages), and anti-CD11c (monocytes/macrophages) (Biolegend, USA) antibodies and using the LIVE/DEAD Fixable Dead Cell Stain Kit (Molecular Probes, USA) at 4°C for 30 min. The cells were then washed with PBS, fixed with Intracellular (IC) fixation buffer (eBioscience, USA) for flow cytometry analysis. The cells were subsequently analyzed using a CytoFLEX S Flow Cytometer (Beckman Coulter, USA).

### ECi (Ethyl Cinnamate)-Based Tissue Clearing

LysM-GFP and CX3CR1-GFP mice were used for visualization of the immune cells, and FSD-647 *Lycopersicon esculentum* (*L. esculentum*) lectin (BioActs, Korea) was intravenously injected to label glycocalyx in the basal membrane of the endothelial cells. ECi clearing method was selected as it has benefits such as minimizing sample shrinkage and preserving endogenous fluorescence for fluorescence imaging (Klingberg et al., 2017). In addition, the method is not toxic, and has been approved by the U.S. Food and Drug Administration (FDA). The aqueous buffer clearing method was excluded as it can make samples inadequately clear and may cause the samples to swell giving them a jelly-like texture. Before conducting tissue clearing using the ECi solution, the mice were perfused with 1X PBS and 4% formaldehyde via the myocardial route. Perfusion using this method enables effective fixation of mice lungs by utilizing the circulatory system of the animal. The intact lungs were then fixed in 4% formaldehyde solution for 2 h and dehydrated in alkaline solutions for 12 h (50/70/100% ethanol, 4 h each). Finally, the samples were cleared using the ECi solution (Alfa Aesar, USA) for 30 min at room temperature. All incubation processes were conducted away from light. The cleared samples were stored in polypropylene tubes till the imaging was done. Light sheet fluorescence microscopy (LFMS) was performed using UltraMicroscope II (LaVison BioTec, Germany). The samples were fixed with and immersed in absolute ECi solution in the mounting chamber during the imaging. Dual side illumination was used, and each plane was obtained every 7 μm. Imaris software (Bitplane, Switzerland) was used to analyze the whole-organ 3D images. The cell counts, volume and area of the granulomas were measured.

### Statistical Analysis

The cell count data were described as either mean ± standard deviation (flow cytometric studies) or mean ± standard error of the mean (histopathological and immunofluorescence studies, and tissue clearing). Prism version 7 (GraphPad Software, USA) was used for statistics and Student's *t*-test was used for analysis.

## Results

**Figure 1:**
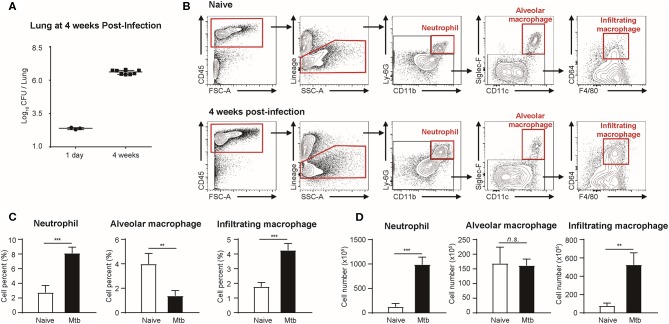
Quantitative analysis of immune cell population in naïve and Mtb (*Mycobacterium tuberculosis*)-infected mice lungs (4 weeks post-infection). **(A)** colony-forming units (CFU) 1 h after (T = 1 day, *n* = 3) and 4 weeks after (T = 4 weeks, *n* = 4) infection with Mtb. **(B)** Gating strategy of immune cells. **(C)** The proportion (%) and **(D)** the absolute count (×10^5^) of different subtypes of immune cells were quantified and compared between naïve and Mtb-infected groups [four mice per group, mean ± standard deviation (SD)]. Statistical analysis was performed using the Student's *t*-test. **P < 0.01, ***P < 0.005. n.s., not significant.

Flow cytometric studies were performed in order to quantify three types of immune cells: neutrophils, alveolar macrophages, and infiltrating macrophages, in both naïve and Mtb-infected mice lungs (Figure 1). All the Mtb-infected mice used in this study were in their fourth week post-infection, at which point the total CFU of the Mtb (T = 4 weeks) showed a higher increase than that at the first day of infection (T = 1 day) (Figure 1A). As described in Figure 1B, flow cytometric studies of both naïve and Mtb-infected mice lungs were performed to compare the population of neutrophils (CD45^+^ and Ly6G^+^), alveolar macrophages (CD45^+^ and Siglec-F^+^), and infiltrating macrophages (CD45^+^ and CD64^+^) between two groups. The proportion of the immune cells showed a notable difference between two groups (Figure 1C). In the naïve group, resident alveolar macrophages (CD45^+^ and Siglec-F^+^) was the predominant cell type (3.97%). Neutrophils and infiltrating macrophages represented 2.74% and 1.77% of the cell population, respectively. In contrast, neutrophils were the most abundant cell type in Mtb-infected lung (8.10%). This was followed by infiltrating macrophages (4.26%), while the proportion of alveolar macrophages was relatively insignificant (1.37%). The actual cell count of alveolar macrophages, however, showed no difference between two groups (Figure 1D). Instead the total cell number in the infected group was larger, making the relative proportion of alveolar macrophages smaller. Both neutrophils and infiltrating macrophages were drastically increased in the infected lung. The mean number of neutrophils was nearly 7 times larger than that of the uninfected lungs, and the mean number of infiltrating macrophages was more than 5 times larger than that of the uninfected lung. These data suggest that both neutrophils and macrophages mediate the host immune reaction against Mtb infection.

**Figure 2:**
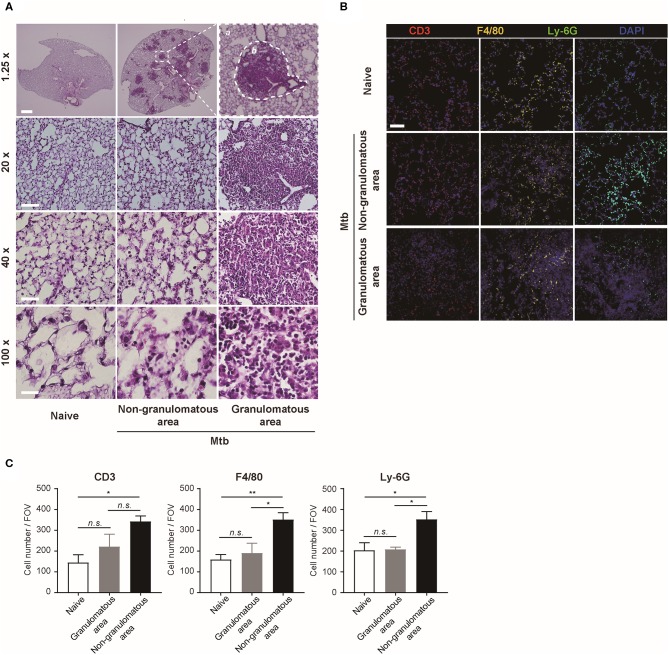
Microscopic morphology and immune cell distribution in naïve and Mtb-infected mice's lungs **(A)** Hematoxylin and eosin (H&E) staining of naïve and Mtb-infected mice lungs (Scale bar, 1.25X: 1 mm, 20X: 100 μm, 40X: 50 μm, 100X: 20 μm). The magnified image contains a single granulomatous area (b) separating it from the non-granulomatous area (a) via a dotted line. **(B)** Immunofluorescence staining of naïve and Mtb-infected mice lungs to verify specific immune cell subtypes. Scale bar, 50 μm. **(C)** The cell counts were compared between the naïve and Mtb-infected group, including granulomatous and non-granulomatous area. Manual counting of each of the immunofluorescence-stained sections was performed [three fields per section, three sections per mouse, three mice per group, mean ± standard error of mean (SEM)]. Statistical analysis was performed using the Student's *t*-test. *P < 0.05, **P < 0.01. Field of view (FOV): 10.24 mm^2^. n.s., not significant.

The morphology and the immune cell population were compared between the naïve and Mtb-infected mice lungs (Figure 2). At low magnification, the whole lung section could be viewed (Figure 2A, upper panel). The granulomas were well-demarcated from the surrounding tissue. At higher magnification, the granulomas showed a highly cellular lesion lacking the normal alveolar structure seen in the naïve lung (Figure 2A). This is consistent with a previous finding that granulomas in C57BL/6 mouse are exclusively cellular without any necrosis or hypoxia (Medina and North, 1998). In order to classify the types of cells in this highly cellular structure, immunofluorescence (IF) microscopy was performed (Figure 2B). Neutrophils, macrophages, and T lymphocytes were distinguished and counted. Since the lung samples were not perfused, most of the immune cells in the naïve mice were located within the alveolar vessels and the non-granulomatous area of the infected mice showed a similar distribution. In contrast, both neutrophils and macrophages were dispersed irregularly throughout the granuloma. The cell number of each subtype of immune cells was counted (Figure 2C). A slight difference in the immune cell population was observed between the granulomas and naive mice lungs, but the difference was not significant (Figure 2C). However, compared with the non-granulomatous area, the infected group contained a larger number of all three subtypes of immune cells than the naïve group (Figure 2C). As stated above, both H&E and immunofluorescence images reveal a limited view of the organ, as they are 2D sectioned images. The sectioned view represents only a part of the whole lesion, making it challenging to quantitatively compare normal and pathologic conditions.

**Figure 3:**
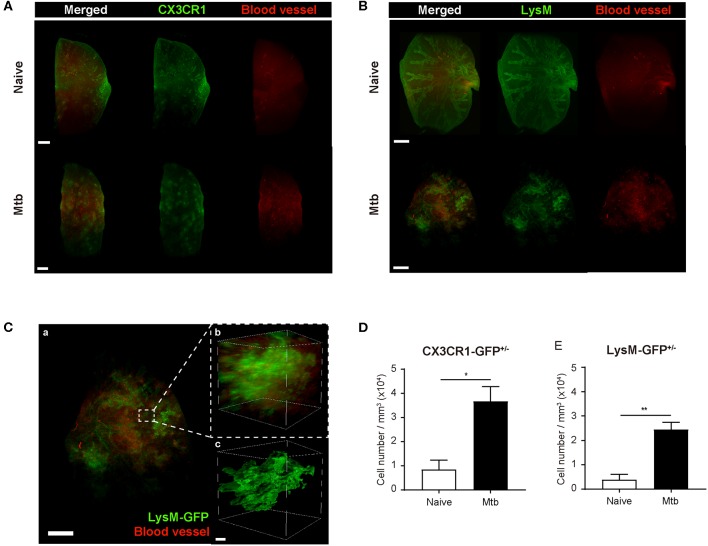
Three-dimensional visualization of the cleared naïve and Mtb-infected mice lung. **(A,B)** Granulomas were clearly detected using CX3CR1-GFP (green fluorescent protein) and LysM-GFP mice. The formation of the blood vessels around the granulomas was also observed. **(C)** The zoomed-in image, containing a single granuloma, was reconstructed as an image of a graphic mass, representing the granuloma, and spots, representing the fluorescent immune cells, for quantitative analysis. **(D,E)** The number of macrophages and neutrophils per volume was compared between naïve and infected group (three granulomas per mouse, three mice per group, mean ± SEM). Statistical analysis was performed using the Student's *t*-test. ^*^P < 0.05, ^**^P < 0.01.

To overcome the limit of 2D analytical methods, 3D whole-lung imaging was performed in each of the cleared samples (Figure 3). In order to observe macrophages, the fluorescence was obtained using CX3CR1-GFP mice that produce green fluorescence protein (GFP) largely in macrophages (Jung et al., 2000) (Figure 3A and Supplementary Videos 1, 2). In order to visualize neutrophils, LysM-GFP mice that has GFP in their neutrophils were used (Faust et al., 2000) (Figure 3B and Supplementary Videos 3, 4). As the lung tissue itself has an auto-fluorescence (Ramanujam, 2000), the structures of the lungs such as the pleura or the bronchus could be identified along with the fluorescent cells (Figures 3A,B and Supplementary Videos 1–4). Multiple granulomas, whose intact shape and size were preserved, were detected in the infected lungs and their distribution in the entirety of the lungs could be visualized (Figures 3A,B). Both neutrophils and macrophages were aggregated near the granulomas. Using zoomed-in images, a single granuloma could be selected and reconstructed into a graphic mass (green), whose volume could be measured (Figure 3C and Supplementary Video 5). Since the fluorescence of the background tissues could be deleted by modulating the field of intensity, the cellular components could be separately selected. The cells were marked as spots (green; Figure 3C). We analyzed the cell counts, specifically those within the borders of the granuloma in the infected samples and divided it by the volume of each granuloma. The number of macrophages were significantly higher in the Mtb-infected granuloma (Figure 3D). In addition, the number of neutrophils were higher in the infected group than the naïve group (Figure 3E). These results were similar to that obtained by the flow cytometric studies. However, since the cell count was limited to the granulomas, this result is more reliable in regard to the analysis of the granulomatous lesions.

To map the vascular distribution of the organ, florescent-labeled *L. esculentum* lectin was infused via the retro-orbital vein to visualize blood vessels prior to sample preparation. It has been reported that uncontrolled angiogenesis occurs in the lungs following Mtb infection (Cronan et al., 2015; Oehlers et al., 2015). Consistent with this report, the vascular distribution differed between the naïve and Mtb-infected mouse's lungs (Figure 3). In the naïve mouse's lung, the vasculature was distributed evenly over the entire organ, showing typical alveolar structures (Figures 3A,B, upper right panels). However, in the Mtb-infected lungs, the vessels showed a more concentrated distribution around the granulomas (Figures 3A,B, lower right panels). In a zoomed-in image of a granuloma (Figure 3C), the vessels appeared to form an outer layer of the lesion, which suggests that the angiogenesis around the granulomas can cause a difference in vascular distribution.

## Discussion

In this study, we employed a tissue-clearing method using ECi solution that preserves the fluorescence of fluorescent proteins, such as GFP, while making an intact organ transparent (Klingberg et al., 2017). Through this procedure, we were able to generate 3D images of the whole mouse lungs containing granulomatous lesions. We demonstrated the distribution of immune cells within each of the selected granulomas and performed a quantitative analysis comparing the immune cell population between Mtb-infected and naïve mouse lungs. The novelty of this study lies in its innovative approach to visualize the immune reactions during TB infection. Compared to the conventional methods such as H&E staining, FACS (fluorescence-activated cell sorting), clearing of the whole lung has a clear advantage of visualizing how the immune cells with fluorescence are located within the pathologic lesion. This allows an accurate counting of specific subtypes of immune cells, which is limited using 2D-sectioned images. In addition, since lung clearing uses the whole lung sample, macroscopic analysis, e.g., shape, volume, distribution of granuloma, can be performed simultaneously with the microscopic analysis. This method can be used in a variety of studies aiming to interpret or modulate the immune response against Mtb infection and should be further evaluated for its application in studying other diseases. For 3D images for the tissue-cleared lungs captured by light sheet microscopy, we employed low magnitude objective lens to acquire the larger area imaging including multiple granulomas, which may result in low-resolution images. To avoid a bias of manual counting of cell numbers from the image data, we performed cell counting by the Imaris software depending on the size and the intensity of the fluorescent cells. Although we just used only 4 weeks period of TB infection for immune cells counting in this study, as an innovative modality to visualize and quantify the immune reactions in TB infection, the whole lung clearing could be a versatile tool to find better clue for therapeutics against TB in combination with other experimental techniques.

## Data Availability Statement

All datasets generated for this study are included in the article/Supplementary Material.

## Ethics Statement

The animal study was reviewed and approved by Yonsei University College of Medicine.

## Author Contributions

G-YK and H-JR performed the experiments and wrote the manuscript. H-HC provided essential reagents for the experiments. SS and Y-MH conceived the study and wrote the manuscript.

## Conflict of Interest

The authors declare that the research was conducted in the absence of any commercial or financial relationships that could be construed as a potential conflict of interest.

## Supplementary Material

Supplementary Video 1 3D clearing imaging of naïve CX3CR1-GFP^+/−^ mouse's lung. Green and red fluorescence represents CX3CR1^+/−^ cells and blood vessels, respectively.

Supplementary Video 2 3D clearing imaging of Mtb-infected CX3CR1-GFP^+/−^ mouse's lung. Green and red fluorescence represents CX3CR1^+/−^ cells and blood vessels, respectively. Blood vessels are aggregated around the granulomas.

Supplementary Video 3 3D clearing imaging of naïve LysM-GFP^+/−^ mouse's lung. Green and red fluorescence represents LysM-GFP^+/−^ cells and blood vessels, respectively.

Supplementary Video 4 3D view of Mtb-infected LysM-GFP^+/−^ mouse's lung. Green and red fluorescence represents LysM-GFP^+/−^ cells and blood vessels, respectively. Blood vessels are clumped around the granuloma lesion.

Supplementary Video 5 A single granuloma of Mtb-infected LysM-GFP^+/−^ mouse's lung. For analysis, a single granulomatous area was reconstructed into a graphic mass, and volume could be measured, also LysM-GFP^+/−^ cells could be defined as green dots.

## Abbreviations

CFU: colony-forming units
ECi: ethyl cinnamate
FACS: fluorescence-activated cell sorting
FBS: fetal bovine serum
FDA: U.S. Food and Drug Administration
GFP: green fluorescent protein
H&E: hematoxylin and eosin
LFMS: light sheet fluorescence microscopy
Mtb: Mycobacterium tuberculosis
OADC: oleic acid albumin dextrose catalase
PBS: phosphate-buffered saline
TB: tuberculosis
WHO: World Health Organization.
